# Response to Dabrafenib and Trametinib of a Patient with Metaplastic Breast Carcinoma Harboring a BRAF V600E Mutation

**DOI:** 10.1155/2020/2518383

**Published:** 2020-03-06

**Authors:** Takuji Seo, Emi Noguchi, Masayuki Yoshida, Taisuke Mori, Maki Tanioka, Kazuki Sudo, Akihiko Shimomura, Kan Yonemori, Yasuhiro Fujiwara, Kenji Tamura

**Affiliations:** ^1^Department of Breast and Medical Oncology, National Cancer Center Hospital, 5-1-1, Tsukiji, Chuo-ku, Tokyo 104-0045, Japan; ^2^Department of Pathology and Clinical Laboratories, National Cancer Center Hospital, 5-1-1, Tsukiji, Chuo-ku, Tokyo 104-0045, Japan

## Abstract

**Background:**

Metaplastic breast carcinomas are rare and carry poor prognoses. They are also more aggressive than other breast cancers and are known for their resistance to chemotherapy. Prolonged treatment with dabrafenib and trametinib is a therapy for malignant melanoma that improves the progression-free survival and overall survival. Such molecular-targeted therapies are also being developed for cancers with BRAF mutation, a driver of malignant melanoma. *Case Presentation*. A 57-year-old woman with metaplastic breast cancer and chemotherapy-refractory massive pleural effusion. After contained anthracycline regimen failure, her breast cancer progressed to an advanced stage. We ordered next-generation sequencing- (NGS-) based tumor molecular profiling from core needle biopsy of the breast. The NGS report indicated the presence of a BRAF V600E mutation. After initiation of dabrafenib and trametinib, her symptom and the pleural effusion were decreased. The first assessment of CT scans showed a decreased pleural effusion and shrunken subcutaneous lesions. Approximately 2 weeks later, a new lesion appeared. She died from 12 weeks after initiation of dabrafenib and trametinib treatment.

**Conclusion:**

To the best of our knowledge, this is the first report of BRAF mutation breast cancer treated with dabrafenib and trametinib and it heralds the possibility of targeted therapy for rare breast cancers.

## 1. Introduction

Metaplastic breast carcinoma is a rare subtype of breast cancer that consists of cells that have undergone metaplasia, a differentiation from glandular to nonglandular squamous or mesenchymal components [1]. It accounts for 0.2%–5% of all breast cancers. Metaplastic carcinoma is usually negative for hormone receptors and human epidermal growth factor receptor 2 (HER2) and is classified as triple negative breast cancer (TNBC) [2–5]. Due to its rarity and the lack of randomized trials, the treatment approach is usually extrapolated from those for other subtypes of breast cancer. Anthracyclines and taxanes have been the mainstay of chemotherapy for decades. However, patients with metaplastic breast carcinoma are often resistant to these conventional chemotherapies, and their prognosis is poor [4–6].

The BRAF gene encodes a serine-threonine protein kinase that plays an important role in the RAS-RAF-MAPK pathway. BRAF mutations are present in many tumor types including cutaneous melanomas (50%), thyroid cancer (20%–50%), colorectal cancer (10%), non-small-cell lung cancer (2%–4%), and hairy cell leukemia (>90%) [7]. The somatic point mutation of BRAF exon-15 (V600E) is the most common mutation among the BRAF gene mutations [7]. BRAF inhibitor monotherapy is usually not long lasting due to the development of acquired resistance and the reactivation of the mitogen-activated protein kinase (MAPK) pathway [8]. Therefore, a combination therapy with a BRAF and a MEK inhibitor would be expect to delay the development of MAPK-driven acquired resistance. Phase III studies on the combination of a BRAF inhibitor (dabrafenib) and a MEK inhibitor (trametinib) in untreated patients with BRAF V600E/V600K-mutated metastatic melanomas demonstrated a prolongation of both the progression-free survival (PFS) and the overall survival (OS) compared to monotherapy with a BRAF inhibitor (vemurafenib or dabrafenib) [9–11]. BRAF inhibition has also been assessed in BRAF-mutant nonmelanoma cancers and is expected to provide new therapeutic options.

We present here the first report of a patient with a BRAF V600E mutation and metastatic metaplastic breast carcinoma that was resistant to anthracycline chemotherapy and was treated with a combination therapy of dabrafenib and trametinib.

## 2. Case Presentation

A previous healthy 57-year-old woman presented to the local hospital with complaints of swelling and right breast pain. Physical examination identified a 5.2 × 4.0 cm induration and redness of the skin in the upper inner quadrant of the right breast with lymphadenopathy. Mammography and ultrasonography revealed no definite tumor, but thickening of the parenchyma was observed throughout the mammary gland. Magnetic resonance imaging revealed a mass enhancement in the upper lesion of the right breast and a tumor invading the pectoralis major muscle. Histopathological examination of the core needle biopsy specimen showed round, pleomorphic, or spindle sarcomatoid cells with myxoid stroma. The differential diagnoses included metaplastic carcinoma with melanocytic differentiation or malignant melanoma. Estrogen and progesterone receptors (ER/PgR) and HER2 expression by immunohistochemistry (IHC) were all negative. The tumor was also negative for pan-cytokeratin (AE1/3), desmin, epithelial membrane antigen (EMA), HHF35, and S100 and positive for CK7 (focal) and melan A (diffuse, strong). From these negative markers, the pathologist ruled out sarcoma and malignant melanoma, and the final pathological diagnosis was metaplastic breast cancer. We cannot show the pictures of the immunohistochemistry results because these tests were performed prior to her referral to our hospital. The patient was tentatively diagnosed as having breast cancer (T4dN3bM0, Stage IIIC) and received 2 cycles of FEC (fluorouracil 500 mg/m^2^, day 1, epirubicin 100 mg/m^2^, day 1, cyclophosphamide 500 mg/m^2^, day 1; every 3 weeks). However, a computed tomography (CT) scan showed progressive disease (PD) of the breast tumor mass and lymph nodes.

**Figure 1:**
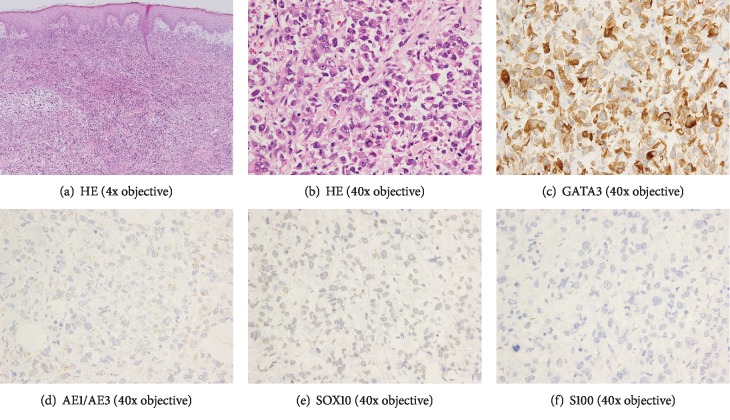
HE and IHC of histopathology. Histopathology of biopsy of the breast. (a) and (b) show HE staining. Poorly differentiated malignant cells invading stromal areas. (c) shows an IHC positive for AE1/3, which rules out sarcoma. (d)–(f) show negative IHCs for GATA3, SOX100, and S100. These results exclude malignant melanoma.

She was referred to our hospital for further treatment. We performed again a biopsy of the breast tumor. Histopathological examination showed a poorly differentiated carcinoma negative for ER, PgR, HER2, gross cystic disease fluid protein, and GATA-binding protein 3 (GATA3) (Figures 1(a)–1(c)). We excluded a diagnosis of malignant melanoma based on the following IHC findings: positive for pankeratin AE1/3 (Figure 1(d)) and negative for CK8, SOX10 (Figure 1(e)), S100 (Figure 1(f)), melan A, CD45, and desmin. Therefore, she was diagnosed as presenting a metaplastic carcinoma with melanocytic differentiation. We checked gene mutation using the tissue sample obtained from prior hospital, which was ineffective for chemotherapy. A targeted next-generation sequencing (NGS) using the NCC Oncopanel System [12] identified genomic alterations including BRAF p.V600E, PIK3CA p.H1047R, CDKN2A p.R58X, and TP53 p.W146X mutations (Table 1). The NCC oncopanel covered 114 gene alternations, along with rearrangements and fusions of 12 oncogenes. Tumor progression after chemotherapy is eligible for NGS using NCC oncopanel in our institute. In this case, we analyzed the tumor sample after anthracycline- and taxane-based chemotherapy. We confirmed the BRAF V600E mutation using a real-time PCR gene mutation assay (THxID™ BRAF kit).

**Figure 2:**
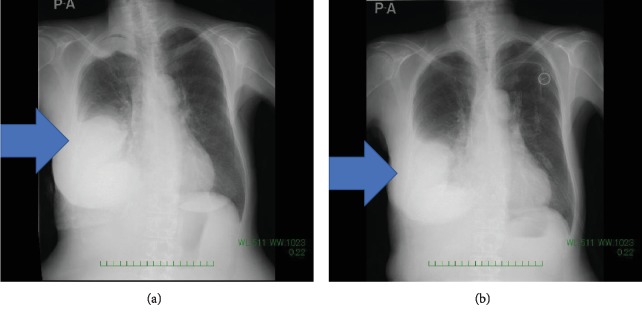
(a) CXR before dabrafenib and trametinib initiation. (b) 1 week after treatment. Arrow shows pleural effusion.

**Figure 3:**
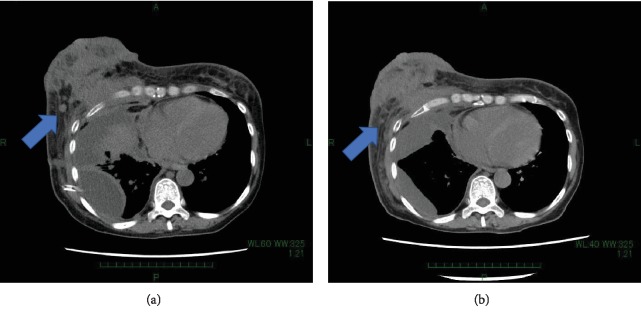
(a) Breast sections of CT scan before initiation of dabrafenib and trametinib. (b) Same section 2 weeks after treatment initiation. The arrow points to reduced mass and decreased pleural effusion.

**Figure 4:**
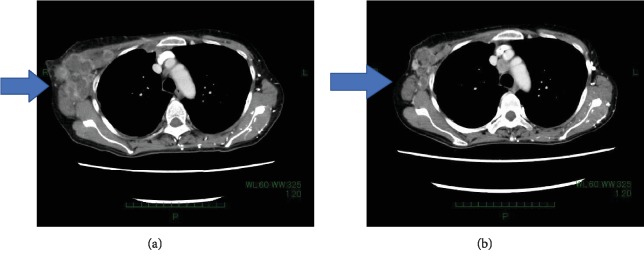
(a) CT scan section showing right axillary lymph nodes before treatment. (b) CT scan section showing right axillary lymph nodes 6 weeks after treatment initiation. Arrow shows right axillary lymph nodes being decreased after treatment.

She underwent palliative radiotherapy to the right breast with a total dose of 30 Gray in 10 fractions; however, the breast mass increased in size and new metastases in the sacrum, causing pleural effusion, appeared. Thoracotomy tube was inserted into the right chest to control pleural effusion. We confirmed an approval by the institutional committee for off-label drug use and started the patient on treatment with dabrafenib at 150 mg BID and trametinib at 2 mg QD after obtaining the patient's informed consent [10]. After a single dose of dabrafenib and trametinib, she experienced grade 1 fever (Common Toxicity Criteria for Adverse Event ver. xx), which we controlled by withdrawing drug administration for a day. The swelling and pain in the right breast improved remarkably after the initiation of dabrafenib and trametinib. A chest X-ray at the 1-week follow-up showed the right pleural effusion had decreased in size (Figure 2(a)). We removed thoracotomy tube without pleurodesis three days after combination therapy. Following 2 weeks of dabrafenib and trametinib administration, a CT scan showed a decrease in pleural effusion and a reduction in the size of the axillary lymph nodes. There was a general improvement in conditions, and we discharged the patient (Figure 3). After 6 weeks of treatment, a CT scan showed further reduction in the size of the tumors in the right breast, axillary lymph nodes, and pleura (Figure 4). The right chest pleural effusion did not increase, and she did not have dyspnea after treatment initiation. However, a new subcutaneous tumor appeared in the right abdominal wall at this time. To confirm the diagnosis of the newly recognized lesion and to monitor whether additional genetic alterations had occurred, we carried out a biopsy of the new subcutaneous tumor after 7 weeks of treatment. The histopathological findings and NGS results were the same as the ones in the primary breast tumor. After 8 weeks of treatment, we observed disease progression with increased pleural effusion and skin metastases in the abdominal wall. The treatment with dabrafenib and trametinib was discontinued and the patient received palliative care. The patient died from breast cancer 12 weeks after the initiation of the dabrafenib and trametinib treatment.

## 3. Discussion

To the best of our knowledge, this is the first case report of a patient with a BRAF-V600E-mutant breast cancer treated with dabrafenib and trametinib. Even though our patient was refractory to conventional chemotherapy and had a rapidly progressing cancer, she experienced a partial response and we observed dramatic primary tumor shrinkage within 2 weeks following the initial treatment of dabrafenib and trametinib. The patient underwent repeated genomic testing at progression to identify mechanisms of acquired resistance, but we found no additional gene alterations.

During the last two decades, molecular profiling for targeted therapy in breast cancer has been studied but still presents a challenge, especially in cases with metaplastic carcinoma. Microarray-based gene expression profiling has demonstrated that most of metaplastic carcinomas are classified as basal-like molecular subtypes [13]. In a study, researchers classified a subgroup of metaplastic carcinomas as claudin-low subtypes [14] (claudin-low is a molecular subtype), but they identified most of the claudin-low tumors as basal-like before assigning them a specific molecular subtype. In view of reports of epidermal growth factor receptor (EGFR) gene amplification and overexpression in metaplastic carcinoma [15, 16], therapeutics targeting EGFR have been studied, but data supporting the efficacy of EGFR-targeted therapy in metaplastic carcinoma have not been obtained.

Breast cancer genomes have been investigated to introduce precision medicine into the clinical practice. The Cancer Genome Atlas data showed that TP53, PIK3CA, and GATA3 are the most often mutated genes in breast cancer [17]. Genomic profiling of metaplastic carcinoma also demonstrated that the most common alterations are in TP53 and PIK3CA [18, 19]. Of note, the frequency of BRAF mutations in breast cancer is extremely small. Albanell et al. reported that comprehensive genomic profiling of 10,428 metastatic breast cancer cases identified 135 (1.3%) BRAF alterations: amplifications (0.6%), mutations (0.5%), and rearrangements (0.2%), respectively [20]. In a report by the same group, among 115 BRAF-mutated cases with known hormone receptor and HER2 statuses, 63 (55%) were TNBCs [21].

The efficacy of BRAF inhibitors against BRAF-mutant solid tumors has been studied [22, 23]. In a phase I study investigating the efficacy of dabrafenib against solid tumors, twenty-eight patients with BRAF-mutant colorectal cancer, non-small-cell lung cancer, ovarian cancer, or thyroid cancer showed partial responses in cases with nonmelanoma tumors [22]. A phase II trial on dabrafenib and trametinib for V600E-mutated anaplastic thyroid cancer showed a highly objective response rate (69%) [23]. A phase II basket trial on dabrafenib and trametinib for patients with various nonmelanoma cancers harboring BRAF V600E mutations is underway (NCT02034110).

Our patient developed resistance to BRAF and MEK inhibitors relatively sooner than has been reported for nonmelanoma tumors [22, 23].

Studies on the resistance mechanisms for BRAF and MEK inhibitors have shown secondary RAS mutations, including those of NRAS or KRAS, can contribute to the resistance development by increasing MAPK reactivation [24]. We tried to identify different pre- and posttreatment mutations but could not find any differences using our target sequence panel.

Another report has indicated that cooccurring PI3K-mTOR pathway aberrations in BRAF V600-mutated nonmelanoma tumors are associated with PFS and OS reductions and may predict primary resistance to BRAF-targeted therapy [25]. Our patient had BRAF and PIK3CA mutations at the beginning of treatment; in retrospect, this may explain the reason for the low efficacy of BRAF and MEK inhibition in our patient. Combinations of BRAF, MEK, and PI3K-mTOR pathway inhibitors may be more effective in such patients. A phase I trial of BRAF inhibitor vemurafenib and mTOR inhibitor everolimus for advanced cancer is underway (NCT01596140). More studies are needed for a better understanding of response and resistance to BRAF-targeted therapy in patients with BRAF-mutant breast cancer.

## Figures and Tables

**Table 1:** The result of NGS.

Genes	CDS	Amino acid
PIK3CA	Exon21:c.A3140G	p.H1047R
BRAF	Exon15:c.T1799A	p.V600E
CDKN2A	Exon2:c.C172T	p.R58X
TP53	Exon5:c.G438A	p.W146X

## Abbreviations

NGS: Next-generation sequencing
CT: Computed tomography
HER2: Human epidermal growth factor receptor 2
EGFR: Epidermal growth factor receptor
PFS: Progression-free survival
OS: Overall survival
ER: Estrogen receptor
PgR: Progesterone receptors
IHC: Immunohistochemistry
EMA: Epithelial membrane antigen
FEC: Fluorouracil+epirubicin+cyclophosphamide
PD: Progressive disease
MAPK: Mitogen-activated protein kinase.
